# Advancing nutritional disorder classification: unleashing the impact of ICD-11 on clinical practice and public health

**DOI:** 10.1017/S0007114524002502

**Published:** 2025-01-14

**Authors:** Meng Zhang, Carine Alsokhn, Robert Jakob, Naishi Li, Yi Wang

**Affiliations:** 1 Collaborating Center for the WHO Family of International Classifications in China, Beijing, People’s Republic of China; 2 Department of Medical Records, Peking Union Medical College Hospital, Chinese Academy of Medical Sciences & Peking Union Medical College, Beijing, People’s Republic of China; 3 Department of Data and Analytics, World Health Organization, Geneva, Switzerland; 4 Department of Endocrinology, Key Laboratory of Endocrinology of National Health Commission, Peking Union Medical College Hospital, Chinese Academy of Medical Sciences & Peking Union Medical College, Beijing, People’s Republic of China

**Keywords:** ICD-11, Nutritional disorders, International Classification of Diseases, Health data standard

Nutritional disorders represent a major global public health issue, with significant and enduring impacts on society and healthcare systems. The International Classification of Diseases (ICD) is fundamental for diagnosing and classifying diseases and health conditions worldwide, supporting health statistics, monitoring, planning and resource allocation. It is extensively used for recording, reporting, researching and comparing nutritional statuses. The prevalence, mortality and disease burden of nutritional disorders are reported based on ICD-coded data^(1–3)^. Studies have demonstrated that nutritional assessment and ICD coding can lead to increased case mix indices, patient complexity and co-morbidity levels, and health insurance reimbursements^(4–6)^.

However, some concepts related to nutritional disorders in ICD-10, which have been in use for over 30 years, are now outdated and do not fully align with current clinical practice and research^(7)^. ICD-11, approved at the 72nd World Health Assembly and effective from 1 January 2022, introduces a range of substantial advancements. These include a semantic network known as the Foundation, updated classifications and terminologies, enhancements in the coding framework, full digital support and interoperability^(8,9)^.

Advised by the Nutrition Guidance Expert Advisory Group, the Department of Nutrition for Health and Development of WHO has actively engaged in updating the nutritional disorders in ICD-11 to align with WHO’s guidelines for nutrition surveillance and management. As a result, significant updates have been made in the classification of undernutrition from ICD-10 to ICD-11. In ICD-11, undernutrition is now categorised based on anthropometric or clinical standards and deficiencies in specific nutrients. For example, ICD-11 provides distinct codes for underweight, wasting, acute malnutrition and stunting in infants, children and adolescents. ICD-11 is currently available in ten languages, with translations into twenty-five additional languages underway, and more expected in the near future. This language-independent classification standardises the understanding and recording of nutritional disorders globally, facilitating national and international comparisons and research, and enabling the tracking of indicators for the Sustainable Development Goals and the Global Nutrition Monitoring Framework^(10,11)^.

ICD-11 provides greater granularity than ICD-10, offering more detailed and specific categories and subcategories for nutritional disorders. For instance, while ICD-10 does not differentiate between overweight and obesity, ICD-11 assigns distinct codes for overweight and for obesity due to energy imbalance across different age groups. Additionally, while ICD-10 groups all forms of beriberi under a single code, ICD-11 subdivides beriberi into dry and wet forms. Furthermore, ICD-11 introduces Wernicke–Korsakoff syndrome as a distinct entity with separate codes for Wernicke encephalopathy and Korsakoff syndrome, reflecting the disease’s continuum. All entities in ICD-11 can be uniquely identified using a uniform resource identifier (URI) in the Foundation, even those without a statistical code. These updates enhance the accuracy of prevalence data and improve the epidemiological monitoring of these conditions.

ICD-11 enhances clinical reporting with its code combination approach known as cluster coding, to capture detailed clinical information through postcoordination^(12)^. For instance, a patient with polyneuropathy due to vitamin B_12_ deficiency would be coded as 8C03.3/5B5F, where 8C03.3 indicates polyneuropathy in nutritional deficiency and 5B5F identifies the vitamin B_12_ deficiency. This approach allows for the reporting of both the manifestation and the aetiology, thereby improving statistical precision and contributing to public health strategies and resource allocation. Meanwhile, ICD-11 offers an array of extension codes for use in cluster coding to capture more details, such as severity, temporality, anatomy and medication. For example, obesity induced by Olanzapine could be captured using the cluster 5B81.1&XM6GK7, where 5B81.1 represents drug-induced obesity and XM6GK7 indicates Olanzapine as the causative agent. This level of detail is invaluable for refining patient management and devising targeted interventions.

The content model used for ICD-11 allows each entity to include a textual definition and diagnostic criteria^(13,14)^. The textual definition provides the meaning and descriptive features of each entity, while the diagnostic criteria outline the necessary diagnostic information. Definitions from WHO sources have been attached to some nutritional disorders in ICD-11, with more anticipated as international consensus is achieved. ICD-11 has significant potential for standardising diagnoses and improving the quality of data reporting. Continuous efforts are required to fully realise these potential benefits.

ICD-11 continues to evolve with medical and scientific advancements, incorporating input from global users. The WHO has established a maintenance mechanism for ICD-11, supported by an open platform and a transparent, rigorous review process. As of August 2024, users from over seventy countries have submitted proposals to the platform, contributing to the ongoing updates of ICD-11. A total of 125 proposals related to nutritional disorders have been received on the maintenance platform, of which 105 (84 %) have been evaluated and decided upon. The remaining proposals are currently undergoing further review or awaiting triage, including those submitted by the author to enrich the Foundation with nutrition-related concepts. Some of these suggested concepts were reported missing in ICD in academic publications but had not been communicated to the ICD-11 maintenance process^(9,15)^. After 2 years following the official coming into effect of ICD-11, 132 Member States and areas are at various phases of implementation^(16)^. As this global implementation progresses, more suggestions and feedback on ICD-11 are anticipated. The nutrition community can contribute to the ICD-11 by directly engaging with the WHO maintenance platform (https://icd.who.int/dev11) when the need for enhancements is identified.

In summary, ICD-11 represents a significant advancement in the classification of nutritional disorders, introducing innovations that enhance clinical practices and public health outcomes. The increased granularity and standardisation of nutritional disorder classifications in ICD-11 support more targeted management, enhance epidemiological monitoring, facilitate international health data comparisons and bolster global health initiatives aimed at addressing these conditions. As ICD-11 continues to evolve with progress in nutrition science, it will unlock its full potential through active engagement of the nutrition community. The transition from ICD-10 to ICD-11 involved collaboration among all relevant stakeholders. Training within nutrition communities and piloting ICD-11 in practice are important steps for a smooth transition.
